# Iron accumulation in hypothalamus promotes age-dependent obesity and metabolic dysfunction of male mice

**DOI:** 10.1186/s43556-025-00324-0

**Published:** 2025-10-02

**Authors:** Xinyu Wang, Xiaoyue Xiong, Ye Xuan, Wen Tian, Liwei Chen, Zhuo Chen, Yi Zhang, Wei L. Shen, Cheng Hu

**Affiliations:** 1 Shanghai Diabetes Institute, Shanghai Key Laboratory of Diabetes Mellitus, Shanghai Clinical Centre for Diabetes, Clinical Research Center, Shanghai Sixth People’s Hospital Affiliated to Shanghai Jiao Tong University School of Medicine, Shanghai, 200233 China; 2 School of Life Science and Technology, ShanghaiTech University, Shanghai, 201210 China; 3 Department of Endocrinology, Jinzhou medical university, Jinzhou, 121001 China; 4 Department of Endocrinology & Metabolism, Jinshan Branch of Shanghai Sixth People’s Hospital, Shanghai, 201599 China

**Keywords:** Iron accumulation, Age-dependent obesity, Hypothalamus, AgRP neurons, Metabolic dysfunction

## Abstract

With the progression of aging, age-dependent obesity and metabolic disorders have garnered increasing attention, yet their underlying mechanisms remain poorly understood. Dysregulation of iron homeostasis is strongly linked to aging; however, its role in age-dependent obesity remains unclear. As the hypothalamus, a key regulator of energy homeostasis, plays a pivotal role in metabolic regulation during aging, we investigated whether hypothalamic iron accumulation contributes to age-dependent obesity. We first observed elevated iron levels in the hypothalamus of aged mice, particularly in the arcuate nucleus. To test whether reducing iron could mitigate obesity, we intranasally administered the iron chelator deferiprone to aged mice and found that it effectively lowered hypothalamic iron levels and ameliorated metabolic function. Using a ferric ammonium citrate-induced iron overload cell model, we discovered that excess iron triggers mitochondrial dysfunction and oxidative stress, leading to ROS-dependent nuclear translocation of forkhead box protein O1 (FoxO1) and subsequent upregulation of AgRP expression. To confirm this mechanism in vivo, we generated agouti-related peptide (AgRP) neuron-specific transferrin receptor 1(Tfrc) knockout mice and found that reducing iron uptake in these neurons decreased ROS levels, inhibited FoxO1 nuclear translocation, and suppressed AgRP neuronal activity in aged mice. This intervention ultimately protected against age-related obesity and metabolic dysfunction. Our study identifies a critical iron accumulation–ROS–FoxO1–AgRP signaling axis in hypothalamic neurons as a key driver of age-dependent obesity. This study elucidates the broader implications of iron homeostasis dysregulation in aging-associated pathologies and offers novel perspectives for investigating age-dependent obesity.

**Supplementary Information:**

The online version contains supplementary material available at 10.1186/s43556-025-00324-0.

## Introduction

Obesity has become a major public health problem [1]. Given the current increase in life expectancy, the prevalence of obesity also raises steadily among older age groups [2, 3]. With advancing age, individuals exhibit an age-related decline in energy expenditure that predisposes to adipose tissue accretion, thereby elevating obesity risk [4, 5]. The increased fat accumulation in elder people elevates the risk of chronic diseases, including insulin resistance, cardiovascular dysfunction, neurodegenerative diseases and osteoarticular disorders, thereby accelerating aging and shortening healthspan [6, 7]. Therefore, the prevention and management of obesity in middle-aged and elderly adults has become an urgent health issue to address in the future. However, the pathogenic mechanisms underlying age-dependent obesity remain largely unclear at present.

It is well known that the hypothalamus plays a central role in maintaining energy homeostasis. The hypothalamic arcuate nucleus (ARC) is situated adjacent to the median eminence (ME), a region with a relatively permeable blood–brain barrier. This unique anatomical positioning enables the ARC to serve as an early sensor of circulating nutrient and hormonal fluctuations, thereby establishing its pivotal role in energy homeostasis regulation [8]. ARC is notably characterized by two functionally distinct neuronal populations, including pro-opiomelanocortin (POMC)-expressing neurons and agouti-related peptide (AgRP)-expressing neurons, which have been extensively demonstrated to play pivotal roles in the regulation of energy homeostasis [9, 10]. The hypothalamic POMC neurons have been reported involved in age-dependent obesity. For example, in hypothalamus of aged mice, Solute carrier family 7 member 14 (SLC7A14) expression in decreased, and overexpression of SLC7A14 in hypothalamic POMC neurons alleviated impaired lipolysis in white adipose tissue of aged mice, thereby alleviating aged-dependent obesity [11]. Genetic ablation of hypoxia-inducible factor 2α (HIF2α) in POMC neurons resulted in progressive, age-dependent-obesity and body weight elevation, concomitant with the development of mild glucose intolerance and insulin resistance [12]. In addition, pharmacological inhibition of the mechanistic target of rapamycin (mTOR) by rapamycin ameliorates age-dependent obesity phenotypes associated with dysregulated mTOR complex 1 (mTORC1) signaling in POMC neurons [13, 14]. However, whether AgRP neurons, which play a crucial role in energy homeostasis, are involved in age-dependent obesity remains unknown.

Iron, an essential trace element, is primarily absorbed in the duodenum following dietary intake and subsequently bound to transferrin for systemic transport via the bloodstream to meet the demands of various tissues. The transferrin-iron complex binds to transferrin receptor 1 (Tfrc) on the cell membrane and enters the cell through endocytosis to fulfill its physiological roles. Excess iron is stored within the cell in the form of ferritin complexes. Meanwhile, iron can be exported out of the cell via the iron exporter ferroportin (FPN1) [15]. Iron plays a critical role in various physiological processes, including oxygen transport, energy metabolism, DNA synthesis and immune defense, making it indispensable for sustaining vital biological functions [16]. However, iron overload can catalyze free radical generation and induce oxidative stress, leading to tissue damage and contributing to the pathogenesis of various diseases [17]. Therefore, systemic iron levels must be tightly regulated to maintain optimal physiological balance and prevent adverse health outcomes. The role of iron in metabolic homeostasis has been extensively reported [18]. The deposition of iron in pancreatic cells can lead to islet dysfunction, resulting in insufficient insulin secretion [19]. Excessive iron accumulation in the liver can disrupt hepatic insulin signaling, leading to lipid deposition and insulin resistance [20]. Meanwhile, reduced iron levels in adipose tissue can ameliorate obesity by influencing intestinal lipid absorption [21]. However, the role of iron in age-dependent obesity remains poorly understood. During aging, the accumulation of iron in various tissues and its role in promoting diseases have garnered increasing attention. Notably, in the brain, iron tends to deposit in regions such as the hippocampus and cortex, thereby contributing to the onset and progression of neurodegenerative disorders [22], including Alzheimer's disease and Parkinson's disease. However, whether iron accumulates in the hypothalamus and contributes to age-dependent obesity in the elderly remains unreported.

In this study, we focused on the critical role of hypothalamic iron accumulation in age-dependent obesity. We discovered that iron accumulates in the hypothalamic ARC of aged mice. Further pharmacological and genetic interventions demonstrated that reducing hypothalamic iron deposition in aging mice effectively suppressed lipid accumulation and prevented obesity. Through in vivo and in vitro experiments, we confirmed that iron promotes AgRP expression via the ROS-FoxO1 signaling pathway, thereby disrupting metabolic homeostasis. Our findings highlight the pivotal role of iron accumulation in AgRP neurons in age-dependent obesity and provide novel insights for potential therapeutic strategies.

## Results

### Hypothalamic iron accumulation during aging

Research has increasingly confirmed that aging contributes to the progressive deterioration of metabolic homeostasis [23]. Consistent with these reports, we demonstrated that compared to 12-week-old mice, 18-month-old mice exhibited significantly increased body weight (Fig. S1a), fat mass (Fig. S1b) and reduced energy expenditure (EE) (Fig. S1c-e), despite comparable food intake (Fig. S1f).

**Fig. 1:**
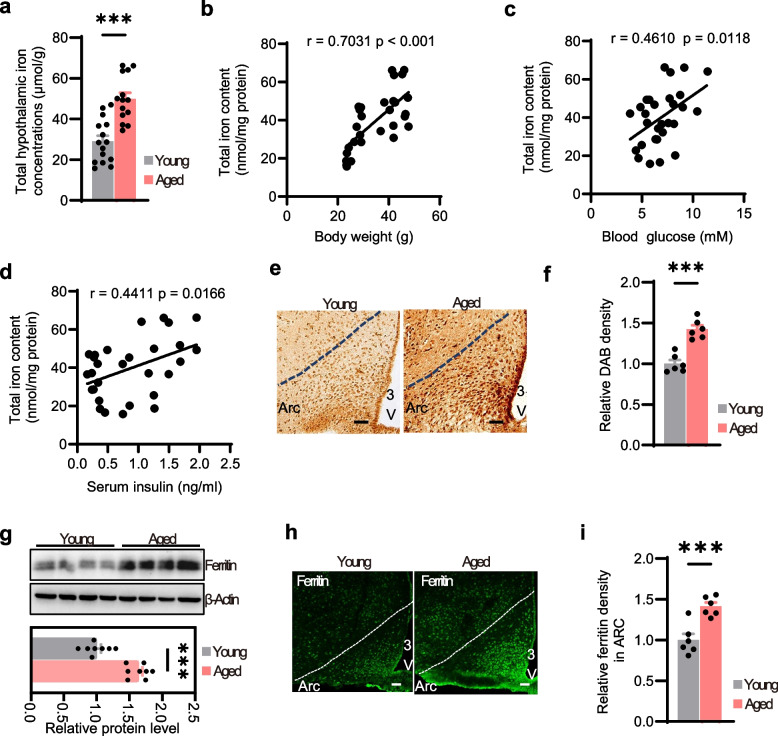
Hypothalamic iron accumulation during aging. **a** Hypothalamic iron levels in young and aged mice as measured by iron assay kit (*n* = 15 for young, *n* = 14 for aged). **b**-**d** Correlation analyses of hypothalamic iron levels with body weight (**b**), blood glucose (**c**), and serum insulin (**d**) (*n* = 29 for each group). r, Pearson correlation coefficient; *P*, *P* value. **e**, **f** Perls’ Prussian blue staining (**e**) and quantification (**f**) of iron deposition in the arcuate nucleus (ARC) of the hypothalamus in young and aged mice (*n* = 6 for each group). **g** Representative western blot showing ferritin expression in the hypothalamus of young and aged mice (*n* = 8 for each group). β-Actin was used as a loading control. **h**, **i** Representative IF staining (**h**) and quantification (**i**) of ferritin in the ARC of young and aged mice (*n* = 6 for each group). Data was presented as the mean ± SEM. ∗ *p* < 0.05, ∗  ∗ *p* < 0.01, ∗  ∗  ∗ *p* < 0.001, two-tailed Student’s t test (**a**, **f**, **g** and **i**). Scale bar, 50 μm

The hypothalamus serves as the center for metabolism regulation, and hypothalamic dysfunction has been implicated in age-dependent metabolic dysfunction [24]. However, the mechanisms underlying hypothalamic dysfunction during aging remain poorly understood. Although a growing body of research has reported that the trace element iron accumulates in multiple organs during aging and contributes to various age-related diseases [16], whether similar iron deposition occurs in the hypothalamus remains unclear. To investigate the relationship between hypothalamic iron level and aging-related metabolic dysfunction, we measured iron level in the hypothalamus of age-dependent obesity mice and young mice. Hypothalamic iron levels were significantly elevated in aged obese mice (Fig. 1a) and positively correlated with body weight, blood glucose, and serum insulin (Fig. 1b–d). Diaminobenzidine (DAB)-enhanced Perls' staining further revealed pronounced iron accumulation specifically within the ARC of aged mice (Fig. 1e, f). The expression of ferritin, which are essential iron storage proteins in mammals, was also markedly increased in the hypothalamus of aged mice (Fig. 1g). Consistent with these findings, immunofluorescence (IF) staining of brain sections confirmed significantly increased ferritin levels in the ARC of aged mice (Fig. 1h, i). Collectively, these results implicated that hypothalamic iron dysregulation is associated with age dependent obesity.

### Intranasal administration of deferiprone (DFP) resulted in a modest but significant reduction in body weight in aged mice

**Fig. 2:**
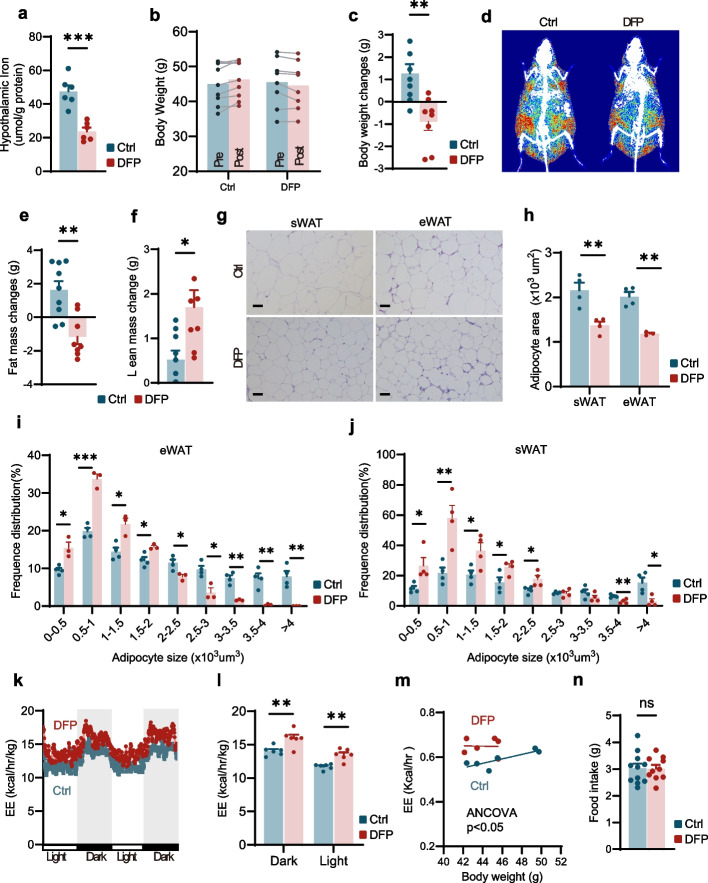
Intranasal administration of deferiprone (DFP) resulted in a modest but significant reduction in body weight in aged mice. **a** Hypothalamic iron levels in aged mice following one month of intranasal administration of DFP (DFP -treated group) or saline (Ctrl group) (*n* = 6 for each group). **b**, **c** Body weight (**b**) and body weight changes (**c**) of aged mice treated for one month with intranasal administration of DFP or saline (*n* = 9 for Ctrl, *n* = 8 for DFP). **d**-**f** Representative DEXA images (**d**), fat mass (**e**), and lean mass (**f**) of aged mice received one month of intranasal administration of DFP or saline (n = 9 for Ctrl, *n* = 8 for DFP-treated). **g**-**j** Representative H&E staining of subcutaneous (sWAT) and epididymal (eWAT) white adipose tissue in aged mice treated for one month of intranasal DFP or saline (**g**) and quantification of adipocyte area (**h**-**j**) (*n* = 4 per group in sWAT; *n* = 4 for Ctrl, *n* = 3 for DFP-treated in eWAT). Scale bar, 50 μm. **k**-**m** Energy expenditure (EE) in aged mice following one month of intranasal administration of DFP or saline (*n* = 6 for each group). **n** Cumulative 24-h food intake in aged mice after one month of intranasal treatment with DFP or saline (*n* = 11 for Ctrl, *n* = 10 for DFP-treated). Data was presented as the mean ± SEM. ∗ *p* < 0.05, ∗  ∗ *p* < 0.01, ∗  ∗  ∗ *p* < 0.001, two-tailed Student’s t test (**a**, **c**, **e**, **f**, **h**, **i**, **j**, **l** and **n**), two-way ANOVA with Bonferroni’s post hoc test (**b**), ANCOVA (**m**)

Based on the observation of iron accumulation in aged mice, we hypothesized whether alleviating iron overload could ameliorate age dependent obesity. To investigate whether hypothalamic iron accumulation contributes to aging-dependent obesity, we employed DFP, an FDA-approved, iron chelator routinely used to alleviate systemic iron burden in thalassemia major and sickle cell patients. DFP forms a 3:1 complex with iron (DFP:Fe), directly targeting intracellular iron pools and exhibits fewer side effects [25]. Intranasal administration allows drugs to bypass the blood–brain barrier and reach the brain directly via pathways such as the olfactory and trigeminal nerves. This approach enhances therapeutic efficacy while reducing drug degradation and avoiding hepatic first-pass effects, making it a non-invasive and efficacious route for delivering drugs to the central nervous system [26]. Therefore, we selected intranasal administration of DFP to reduce iron levels in the hypothalamus. We identified that intranasal administering DFP at the dose of 90 μg per mouse effectively reduced hypothalamic iron content in aged dependent obesity mice to levels comparable to young controls, without altering systemic iron status (Fig. 2a, Fig. S2a). Based on this finding, we employed this optimized dosage in subsequent experiments, treating aging-related obese mice daily for 30 days. Metabolic assessments were performed in both DFP-treated mice and saline-treated mice before and after the intervention.

DFP-treated mice exhibited reduced body weight (Fig. 2b, c) and decreased fat mass (Fig. 2d, e), along with increased lean mass (Fig. 2f). Histological analysis of subcutaneous white adipose tissue (sWAT) and epididymal white adipose tissue (eWAT) revealed smaller lipid droplets in DFP-treated mice (Fig. 2g, h), with a higher frequency of smaller adipocytes compared to controls (Fig. 2i, j). Metabolic monitoring demonstrated significantly enhanced energy expenditure (EE) in DFP-treated mice (Fig. 2k–m) without increased activity (Fig. S2b, c), while food intake remained unchanged (Fig. 2n). To determine whether these metabolic benefits of intranasal DFP administration were specific to aged mice, we treated young mice with DFP and observed no changes in body weight (Fig. S3a, b), fat mass (Fig. S3c), lean mass (Fig. S3d), or EE (Fig. S3e–g). These results demonstrate that iron chelation within the central nervous system via intranasal administration of DFP enhances energy expenditure (EE) and ameliorates metabolic parameters in aged mice.

### Deferiprone (DFP) ameliorated aged-dependent metabolic dysfunction

**Fig. 3:**
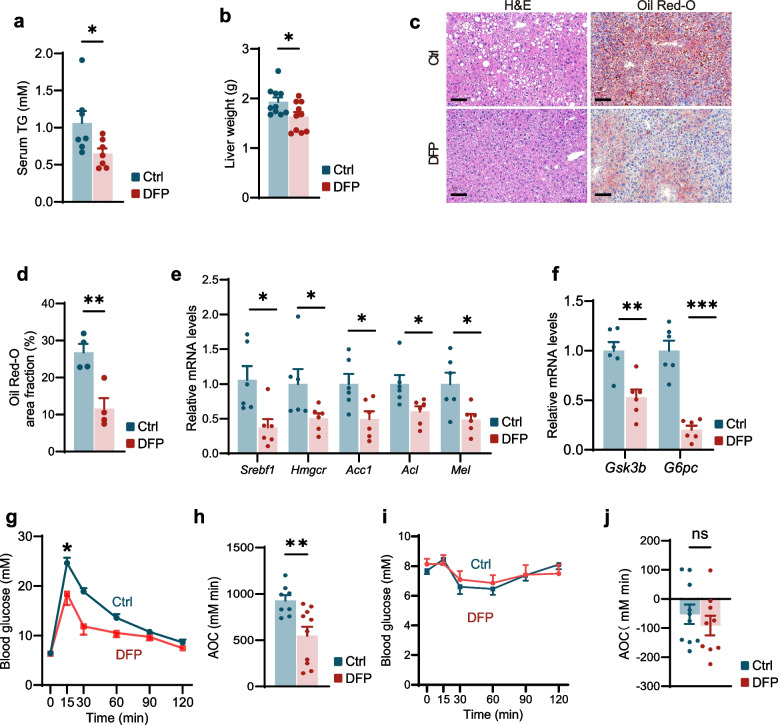
DFP ameliorated aged-dependent metabolic dysfunction. **a** Serum triglyceride (TG) levels in aged mice following one month of intranasal administration of DFP or saline (*n* = 7 for each group). **b** Liver weight of aged mice treated for one month with intranasal DFP or saline (*n* = 10 per group). **c**, **d** Representative images of H&E staining and Oil Red O staining images (**c**) and quantification of lipid droplet area (**d**) in the liver of aged mice after one month of intranasal treatment with DFP or saline (*n* = 4 for each group). Scale bar, 50 μm. **e**, **f** Relative mRNA expression levels of lipogenesis genes (**e**) and glycogen synthesis genes (**f**) in the liver of aged mice receiving one month of intranasal DFP or saline (*n* = 6 per group), as determined by RT–m. **g**, **h** Glucose tolerance test (GTT) (**g**) and area of the curve (AOC) of GTT (**h**) of aged mice received one month of intranasal administration of DFP or saline (*n* = 10 for Ctrl, *n* = 8 for DFP). **i**, **j** Insulin tolerance test (ITT) (**i**) and AOC of ITT (**j**) in aged mice received one month of intranasal administration of DFP or saline (*n* = 10 for Ctrl, *n* = 9 for DFP). Data was represented as mean ± SEM. *p < 0.05, **p < 0.01, ***p < 0.001, two-tailed Student’s t-test (**a**, **b**, **d**, **e**, **f**, **h** and **j**), two‐way ANOVA with Bonferroni’s post hoc test (**g** and **i**)

We further investigated whether the reduction of hypothalamic iron levels in aged mice was accompanied by improvements in other metabolic parameters. Our investigation revealed that intranasal administration of DFP in aged mice significantly reduced serum triglyceride (TG) levels (Fig. 3a) and liver weight (Fig. 3b). Histological analysis via H&E and Oil Red O staining confirmed a reduction in hepatic lipid droplet area (Fig. 3c, d). Molecular analyses demonstrated that DFP-treated mice exhibited significant downregulation of lipogenic genes, including *Srebf1*, *Hmgcr*, *Acc1*, *Acl*, and *Fasn* (Fig. 3e). Furthermore, expression of genes associated with glycogen synthesis (*Gsk3b*) and gluconeogenesis (*G6pc*) were also markedly reduced (Fig. 3f). Collectively, these results demonstrated that reducing central iron levels via intranasal DFP elicits beneficial effects on hepatic metabolism, specifically ameliorating hepatic steatosis.

Aging also disrupted the homeostasis of blood glucose [27]. To determine whether reducing central nervous system (CNS) iron levels could improve aging-related glucose metabolism, we assessed glucose and insulin tolerance in intranasal DFP-treated versus saline-treated aged mice. DFP administration significantly improved glucose tolerance compared to controls (Fig. 3g, h). To rule out confounders such as body weight differences or glycogen depletion, we performed intraperitoneal glucose tolerance tests (GTT) in fasted (5-h) mice, with glucose doses adjusted for lean mass. DFP treatment remained effective under these conditions (Fig. S4a, b). In contrast, insulin sensitivity was unchanged (Fig. 3i, j), possibly due to delayed metabolic adaptation or incomplete targeting of core insulin resistance mechanisms. As expected, DFP administration in young mice had no effect on glucose tolerance (Fig. S4c, d) or insulin tolerance (Fig. S4e, f). Together, these results demonstrate that CNS iron chelation via intranasal DFP alleviates metabolic dysfunction in aged mice.

### Iron regulated the expression of AgRP by promoting mitochondrial oxidative stress

**Fig. 4:**
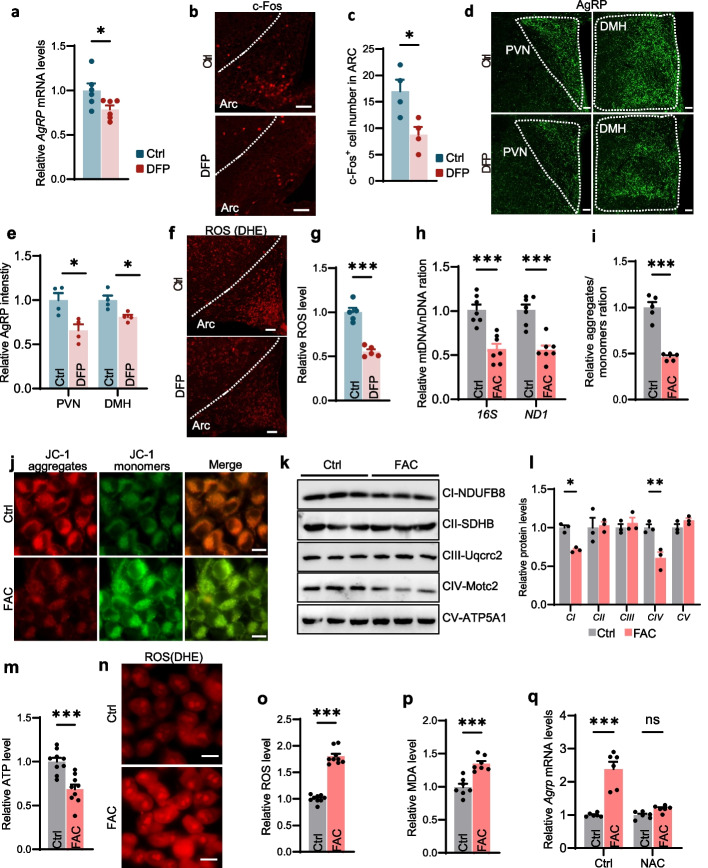
Iron overload regulated the expression of AgRP by promoting mitochondrial oxidative stress. **a** Relative mRNA levels of *Agrp* in the hypothalamus of aged mice following one month of intranasal administration of DFP or saline (*n* = 6 for each group), as measured by RT–qPCR. **b**, **c** Representative immunofluorescence (IF) staining images (**b**) and quantification (**c**) of c-Fos⁺ cells in the ARC of aged mice treated with intranasal DFP or saline for one month (*n* = 4 per group). **d**, **e** Representative IF images (**d**) and quantification (**e**) of AgRP-immunoreactive fibers projecting to the paraventricular nucleus (PVN) and dorsomedial hypothalamus (DMH) (*n* = 4 per group). **f**, **g** Representative dihydroethidium (DHE) staining images (**f**) and quantification (**g**) in the ARC of aged mice after one month of intranasal DFP or saline treatment (*n* = 5 per group). **h** Mitochondrial DNA copy number relative to nuclear DNA (mtDNA/nDNA) in GT1-7 cells treated with ferric ammonium citrate (FAC) or vehicle control (*n* = 7 per group). **i**, **j** Representative JC-1 staining (indicative of mitochondrial membrane potential) images (**j**) and quantification (**i**) of JC-1 red/green fluorescence ratio (*n* = 5 per group). **k**, **l** Western blot analysis (**k**) and quantification (**l**) of electron transport chain (ETC) complex protein levels in GT1-7 cells treated with FAC or vehicle (*n* = 3 per group). **m** Relative ATP levels in GT1-7 cells treated with FAC or control (**n** = 9 per group). **n**, **o** Representative DHE staining images (**n**) and quantification (**o**) of superoxide levels in GT1-7 cells treated with FAC or control (*n* = 9 per group). **p** Malondialdehyde (MDA) levels in GT1-7 cells treated with FAC or control (*n* = 7 per group). **q** Relative Agrp mRNA expression in GT1-7 cells treated with FAC or control, in the presence or absence of NAC co-treatment. (*n* = 6 per group). Data was presented as the mean ± SEM. ∗ *p* < 0.05, ∗  ∗ *p* < 0.01, ∗  ∗  ∗ *p* < 0.001, two-tailed Student’s t test (**a**, **c**, **e**, **g**, **h**, **i**, **l**, **m**, **o**, **p** and **q**). Scale bar, 50 μm

Based on these findings, we sought to elucidate the mechanisms underlying DFP-mediated metabolic improvement in aged mice. Given the pronounced iron accumulation observed specifically in the ARC of aged mice, we hypothesized that neuronal dysfunction in this hypothalamic nucleus drives age-dependent metabolic dysfunction. Since AgRP neurons and POMC neurons in ARC constitute the core circuit for metabolic control [8], we analyzed the gene expression of *Agrp* and *Pomc* in hypothalamus of DFP-treated aged mice. Intranasal DFP administration decreased *Agrp* mRNA expression in ARC of aged mice compared to controls, while *Pomc* mRNA levels remained unchanged (Fig. 4a, Fig. S5a). IF staining confirmed the reduction of c-Fos⁺ neurons number in the medial ARC of aged mice (Fig. 4b, c), where AgRP neurons reside, in DFP-treated mice. AgRP neurons release the neuropeptide AgRP to hypothalamic nuclei such as the paraventricular nucleus (PVN) and dorsomedial hypothalamus (DMH), regulating their functions to reduce EE and promote obesity [8]. Therefore, we examined the expression of AgRP in the DMH and PVN nuclei. The results showed that DFP-treated aged mice exhibited a significant decrease in AgRP expression (Fig. 4d, e). These findings implicated suppressed AgRP neuronal activity and reduced AgRP expression in DFP-treated aged mice.

Oxidative stress is intimately associated with the aging process. The chronic accumulation of mitochondrial-derived reactive oxygen species (ROS) leads to widespread mitochondrial and cellular dysfunction, ultimately resulting in cellular senescence [28]. Notably, persistent intracellular iron accumulation exacerbates this process by inducing oxidative stress and mitochondrial dysfunction [29]. Based on these observations, we hypothesized that iron accumulation during aging in the hypothalamic ARC triggers oxidative stress, which subsequently causes mitochondrial dysfunction and ultimately impairs neuronal function. Using dihydroethidium (DHE) staining, we confirmed that DFP significantly reduced ROS levels in the ARC of aged mice (Fig. 4f, g). To investigate the underlying molecular mechanism, we established an iron overload model in AgRP-expressing GT1-7 cells using ferric ammonium citrate (FAC). FAC treatment induced mitochondrial iron accumulation (Fig. S5b, c), decreased the mitochondrial-to-nuclear DNA ratio (mtDNA/nDNA) (Fig. 4h), and triggered mitochondrial depolarization, as indicated by increased JC-1 monomers and reduced aggregates (Fig. 4i, j). Immunoblotting revealed downregulated protein levels of mitochondrial electron transport chain (ETC) complexes I and Ⅳ in FAC-treated cells (Fig. 4k, l). These cells also exhibited reduced ATP production (Fig. 4m), elevated ROS (Fig. 4n, o), and increased malondialdehyde (MDA) levels (Fig. 4p), confirming iron overload-induced mitochondrial dysfunction and oxidative stress.

Notably, FAC treatment increased *Agrp* mRNA expression in GT1-7 cells, an effect abolished by the antioxidant N-acetylcysteine (NAC) (Fig. 4q), which suppressed oxidative stress. To further validate that mitochondrial-derived ROS mediates iron-dependent AgRP upregulation, we used Mito-TEMPO, a mitochondrial ROS scavenger. Consistent with NAC treatment, Mito-TEMPO significantly attenuated iron-induced AgRP expression (Fig. S5d). Together, these results demonstrate that iron accumulation elevates AgRP expression through mitochondrial dysfunction and oxidative stress.

### Iron regulated AgRP expression through reactive oxygen species (ROS)- forkhead box protein O1(FoxO1) signaling

Having established that iron overload upregulates AgRP expression through oxidative stress, we further investigated how oxidative stress induced by iron accumulation impacts AgRP neuronal function. FoxO1, a transcription factor highly expressed in AgRP neurons, regulates energy balance by controlling *Agrp* transcription [30, 31] and is known to be activated by both iron excess and ROS, which promote its nuclear translocation [31–34].

**Fig. 5:**
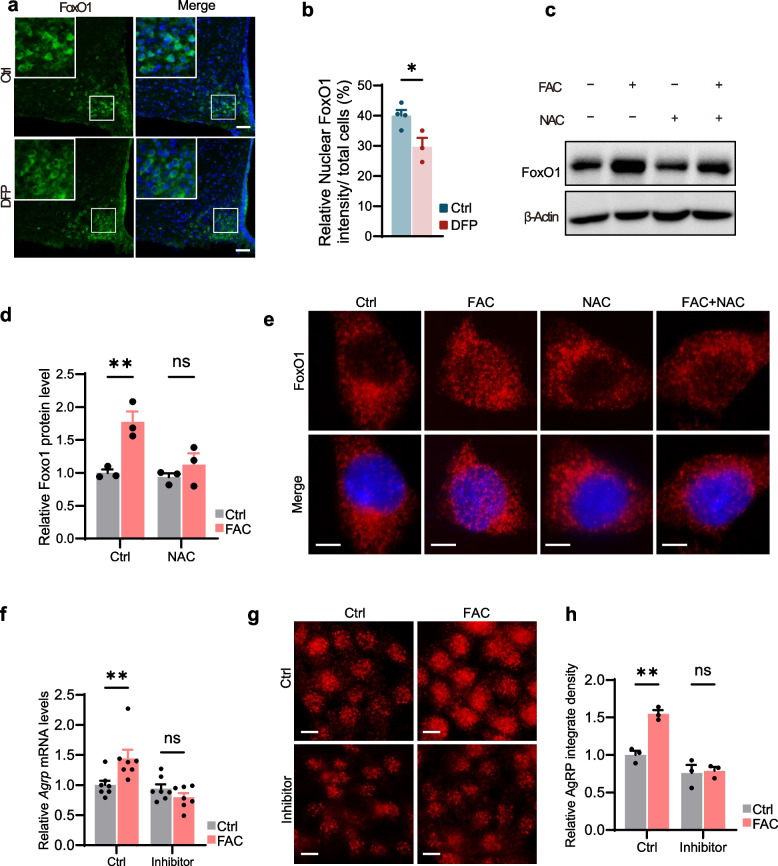
Iron overload regulated AgRP expression through ROS-FoxO1. **a**, **b** Representative IF staining images (**a**) and quantification (**b**) of FoxO1 in the ARC of aged mice after one month of intranasal administration of DFP or saline (*n* = 4 for Ctrl, *n* = 3 for DFP-treated). Cell nuclei were counterstained with DAPI. Scale bar, 50 μm. **c**, **d** Western blot analysis (**c**) and quantification (**d**) of FoxO1 protein expression in GT1-7 cells treated with FAC and NAC (*n* = 3 for each group). β-actin served as a loading control. **e** Representative IF staining images of FoxO1 localization in GT1-7 cells treated with FAC and NAC. Cell nuclei were counterstained with DAPI. Scale bar, 20 μm. **f** Relative Agrp mRNA levels in GT1-7 cells treated with FAC and FoxO1 inhibitor AS1842856, as measured by RT–qPCR (*n* = 7 for each group). **g**, **h** Representative IF staining images (**g**) and quantification (**h**) of AgRP in GT1-7 cells treated with FAC and FoxO1 inhibitor AS1842856. Scale bar, 10 μm. (*n* = 3 for each group). Data was presented as the mean ± SEM. ∗ *p* < 0.05, ∗  ∗ *p* < 0.01, ∗  ∗  ∗ *p* < 0.001, two-tailed Student’s t test (**b**, **d**, **f** and **h**)

IF staining revealed significant cytoplasmic retention of FoxO1 in the ARC of aged mice following intranasal DFP administration (Fig. 5a, b), suggesting reduced nuclear activity. In GT1-7 cells, FAC treatment increased FoxO1 expression and promoted its nuclear accumulation, as demonstrated by immunoblotting and IF staining (Fig. 5c-e). This effect was reversed by the antioxidant N-acetylcysteine (NAC) (Fig. 5c-e). Importantly, pharmacological inhibition of FoxO1 with AS1842856 substantially attenuated the FAC-induced increase in *Agrp* mRNA levels (Fig. 5f) and AgRP protein expression (Fig. 5g, h). Together, these findings demonstrate that iron overload triggers mitochondrial ROS production, which activates FoxO1 expression and nuclear translocation to stimulate *Agrp* transcription.

### The specific deletion of transferrin receptor (Tfrc) in AgRP neurons inhibited ROS-Foxo1 signaling

**Fig. 6:**
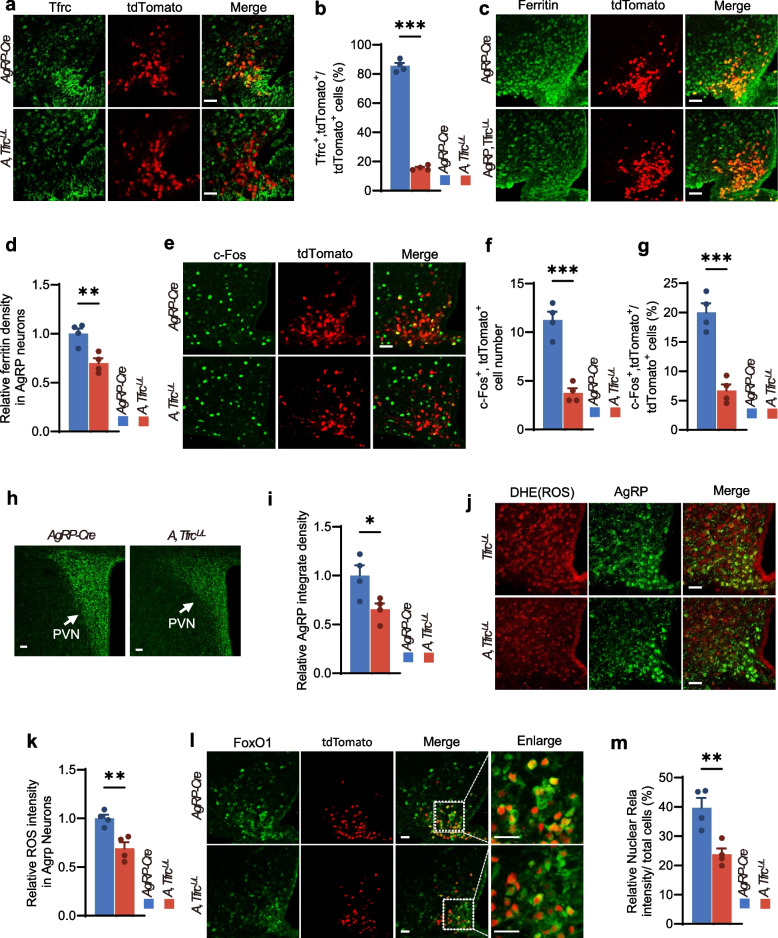
The specific deletion of Tfrc in AgRP neurons inhibited ROS-Foxo1 signaling. **a**, **b** Representative IF staining images (**a**) and quantification (**b**) of Tfrc in the ARC of *AgRP-Cre* and *A, Tfrc*^*L/L*^ mice. AgRP neurons were labeled in red. (*n* = 4 per group). **c**, **d** Representative IF staining images (**c**) and quantification (**d**) of ferritin in the ARC of *AgRP-Cre* and *A, Tfrc*^*L/L*^ mice (*n* = 4 per group). **e**–**g** Representative IF staining images (**e**) and quantification (**f**, **g**) of c-Fos in the ARC of *AgRP-Cre* and *A, Tfrc*^*L/L*^ mice (*n* = 4 per group). **h**, **i** Representative IF images (**h**) and quantification (**i**) of AgRP fibers projecting to PVN of *AgRP-Cre* and *A, Tfrc*^*L/L*^ mice (*n* = 4 per group). **j**, **k** Representative DHE staining images (**j**) and quantification (**k**) in the ARC of *AgRP-Cre* and *A, Tfrc*^*L/L*^ mice (*n* = 4 per group). **l**, **m** Representative IF staining images (**l**) and quantification (**m**) of FoxO1 in the ARC of *AgRP-Cre* and *A, Tfrc*^*L/L*^ mice (*n* = 4 per group). Data was presented as the mean ± SEM. ∗ *p* < 0.05, ∗  ∗ *p* < 0.01, ∗  ∗  ∗ *p* < 0.001, two-tailed Student’s t test (**b**, **d**, **f**, **g**, **i**, **k** and **m**). Scale bars, 50 μm

To definitively determine whether iron overload mediates AgRP neuronal dysfunction, we genetically reduced iron metabolism in AgRP neurons by delete transferrin receptor (Tfrc). As a membrane-localized protein, Tfrc binds transferrin-bound iron (Tf-Fe), thereby mediating cellular iron uptake. We generated mice with AgRP neuron-specific knockout of the iron uptake rate-limiting gene *Tfrc*. We crossed *AgRP-Cre* mice with *Tfrc*^*LoxP/LoxP*^ (*Tfrc*^*L/L*^) mice to generate *AgRP-Cre, Tfrc*^*LoxP/LoxP*^ (*A*, *Tfrc*^*L/L*^) mice with selective deletion of Tfrc in AgRP neurons. To label AgRP neurons, we crossed *AgRP-Cre* mice with *tdTomato/Ai14* mice. *Tfrc*^*LoxP/LoxP*^ mice and *AgRP-Cre* mice served as controls. IF staining confirmed the knockout efficiency of Tfrc in AgRP neurons (Fig. 6a, b). Correspondingly, ferritin expression in AgRP neurons of *A*, *Tfrc*^*L/L*^ mice reduced significantly (Fig. 6c, d), indicating the iron levels were obviously decreased in AgRP neurons. Furthermore, *A*, *Tfrc*^*L/L*^ mice exhibited reduced c-Fos co-localization with AgRP neurons (Fig. 6e-g) and density of AgRP fibers projecting to the PVN (Fig. 6h, i), which both indicated decreased AgRP neuronal activity. As expected, DHE staining revealed lower ROS levels in the AgRP neurons of *A*, *Tfrc*^*L/L*^ mice compared to controls (Fig. 6j, k). Immunostaining of FoxO1 also demonstrated increased cytoplasmic retention (reduced nuclear translocation) of FoxO1 in AgRP neurons of *A*, *Tfrc*^*L/L*^ mice (Fig. 6l, m). These results indicated that iron levels in AgRP neurons critically regulate their activity through ROS and related FoxO1 nuclear translocation.

### Transferrin receptor (Tfrc) ablation in AgRP neurons ameliorated age-dependent metabolic dysfunction

**Fig. 7:**
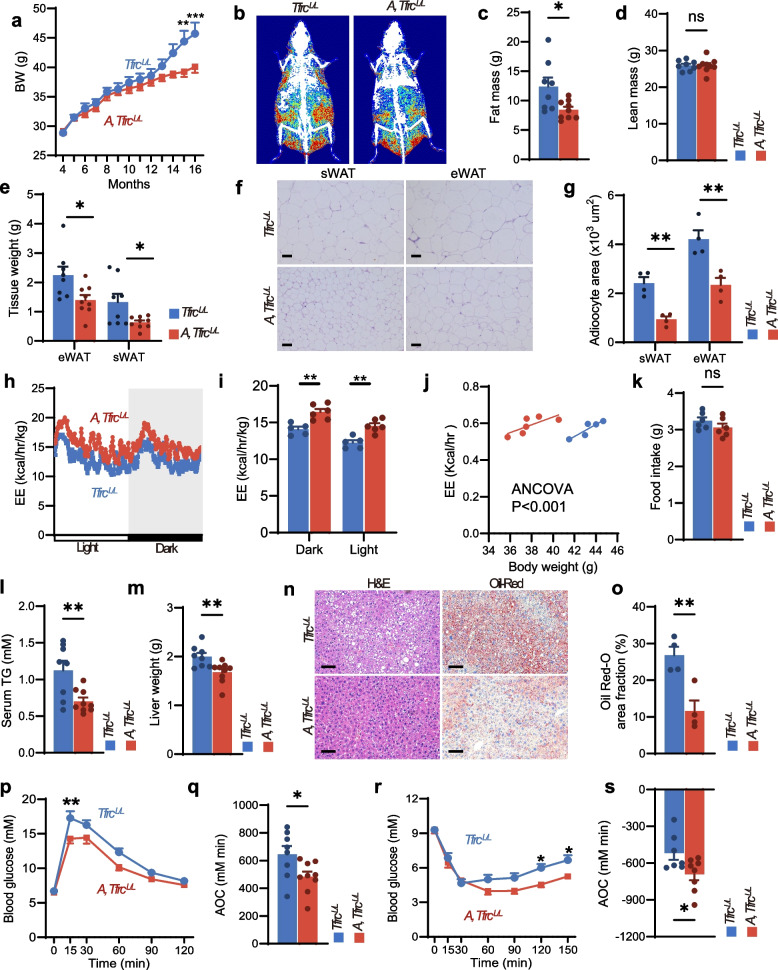
Tfrc ablation in AgRP neurons ameliorated age-dependent metabolic dysfunction. **a**-**d** Body weight (**a**), representative DEXA images (**b**) and body composition (**c**, **d**) of *Tfrc*^*L/L*^ and *A, Tfrc*^*L/L*^ mice fed a CD for 16 months (*n* = 8 for *Tfrc*^*L/L*^, *n* = 9 for *A, Tfrc*^*L/L*^). **e** Mass of eWAT and sWAT of *Tfrc*^*L/L*^ and *A, Tfrc*^*L/L*^ mice after 16 months on a CD (*n* = 8 for *Tfrc*^*L/L*^, *n* = 9 for *A, Tfrc*^*L/L*^). **f**, **g** Representative H&E-stained sections of sWAT and eWAT (**f**) and quantification of adipocyte cross-sectional area (**g**) in *Tfrc*^*L/L*^ and *A, Tfrc*^*L/L*^ mice fed a CD for 16 months(*n* = 4 per group). **h**-**j** EE of *Tfrc*^*L/L*^ and *A, Tfrc*^*L/L*^ mice fed a CD for 16 months (*n* = 5 for *Tfrc*^*L/L*^, *n* = 6 for *A, Tfrc*^*L/L*^). **k** Cumulative 24-h food intake of *Tfrc*^*L/L*^ and *A, Tfrc*^*L/L*^ mice fed a CD for 16 months (*n* = 6 per group). **l** Serum TG levels of *Tfrc*^*L/L*^ and *A, Tfrc*^*L/L*^ mice fed a CD for 16 months (*n* = 8 for *Tfrc*^*L/L*^, *n* = 9 for *A, Tfrc*^*L/L*^). **m** Liver weight of *Tfrc*^*L/L*^ and *A, Tfrc*^*L/L*^ mice fed a CD for 16 months (*n* = 8 for *Tfrc*^*L/L*^, *n* = 9 for *A, Tfrc*^*L/L*^). **n**, **o** Representative H&E staining and Oil Red O-stained liver sections (**n**) and quantification of lipid droplet area (**o**) of *Tfrc*^*L/L*^ and *A, Tfrc*^*L/L*^ mice fed a CD for 16 months (*n* = 4 per group). **p**, **q** GTT (**p**) and AOC of GTT (**q**) of *Tfrc*^*L/L*^ and *A, Tfrc*^*L/L*^ mice after 16 months on a CD (*n* = 8 for *Tfrc*^*L/L*^, *n* = 9 for *A, Tfrc*^*L/L*^). **r**, **s** ITT (**r**) and AOC of ITT (**s**) of *Tfrc*^*L/L*^ and *A, Tfrc*^*L/L*^ mice fed a CD for 16 months (*n* = 7 for *Tfrc*^*L/L*^, *n* = 8 for *A, Tfrc*^*L/L*^). Data was represented as mean ± SEM. *p < 0.05, **p < 0.01, ***p < 0.001, two-tailed Student’s t-test (**c**, **d**, **e**, **g**, **i**, **k**, **l**, **m**, **o**, **q** and **s**), two‐way ANOVA with Bonferroni’s post hoc test (**a**, **p** and **r**), ANCOVA (**j**). Scale bar, 50 μm

To investigate whether iron reduction specifically in AgRP neurons could ameliorate age-dependent obesity and metabolic dysfunction, we analyzed *A*, *Tfrc*^*L/L*^ mice and *Tfrc*^*L/L*^ mice fed a chow diet (CD) until 16 months of age. *A*, *Tfrc*^*L/L*^ mice exhibited significantly lower body weight (Fig. 7a) and reduced fat mass (Fig. 7b, c) compared to controls, while lean mass remained unchanged (Fig. 7d). Histological analysis of eWAT and sWAT revealed decreased adipocyte size (Fig. 7e-g) and a higher frequency of smaller adipocytes compared to controls (Fig. S6a, b) in *A*, *Tfrc*^*L/L*^ mice. Metabolic monitoring demonstrated significantly increased EE in *A*, *Tfrc*^*L/L*^ mice (Fig. 7h-j), with no change in food intake (Fig. 7k).

We next assessed hepatic metabolism and glucose homeostasis in these mice.* A*, *Tfrc*^*L/L*^ mice also displayed improved hepatic metabolism, including reduced serum TG levels (Fig. 7l), decreased liver weight (Fig. 7m), and diminished hepatic lipid droplet area confirmed by H&E and Oil Red O staining (Fig. 7n, o). Furthermore, *A*, *Tfrc*^*L/L*^ mice showed ameliorated glucose tolerance (Fig. 7p, q) and insulin tolerance compared to controls (Fig. 7r, s). Collectively, these findings demonstrated that reduced iron levels within AgRP neurons ameliorates obesity and metabolic dysfunction in aged mice.

## Discussion

Aging predisposes individuals to obesity, partly through hypothalamic dysfunction that disrupts energy homeostasis [35]. AgRP neurons in the hypothalamus play key roles in sensing metabolic signals and regulating feeding [36], yet how aging affects their function remains poorly understood. Here, we show that iron accumulates in the hypothalamic arcuate nucleus of aged obese mice. Reducing hypothalamic iron via intranasal iron chelator DFP suppressed AgRP expression, increased energy expenditure, and improved obesity. Mechanistically, iron overload increased mitochondrial ROS, promoting FoxO1 nuclear translocation and AgRP upregulation. AgRP-specific Tfrc knockout reduced neuronal iron, decreased ROS, inhibited FoxO1, and attenuated AgRP activity, thereby enhancing whole-body energy metabolism. These findings reveal a pathway whereby iron excess in AgRP neurons promotes age-related obesity via ROS–FoxO1 signalling.

Iron dyshomeostasis is a hallmark of aging, leading to iron accumulation in multiple organs including the brain, and is implicated in age-related diseases such as neurodegeneration and metabolic dysfunction [37, 38]. While iron buildup is well-documented in regions like the hippocampus and substantia nigra in Alzheimer’s and Parkinson’s diseases [39, 40], its role in other brain areas remains unclear. Here, we reveal iron accumulation in the hypothalamic arcuate nucleus in age-dependent obese mice. Intranasal administration of the iron chelator DFP reduced brain iron levels without affecting systemic iron, and ameliorated obesity. Given established links between iron chelation and improved outcomes in neurodegenerative disorders [41–43], and the association between obesity and neurodegeneration, intranasal DFP may represent a dual-purpose therapeutic strategy for both conditions.

Mitochondrial dysfunction is a central hallmark of aging, closely linked to oxidative stress through ROS-mediated damage [44–46]. Age-related mitochondrial iron accumulation further exacerbates ROS production, impairing mitochondrial function and promoting cellular decline [47–49]. In this study, iron overload in AgRP neurons induced mitochondrial oxidative stress and increased AgRP expression. In the present study, utilizing an AgRP-expressing cell model, we demonstrated that iron overload significantly impaired mitochondrial function, exacerbated intracellular oxidative stress and consequently upregulated AgRP expression. In vivo experiments in aged mice confirmed that DFP treatment effectively attenuated hypothalamic AgRP expression. AgRP neuropeptides released from AgRP neurons modulate activity in key hypothalamic nuclei including the PVN and DMH, thereby disrupting energy homeostasis and promoting obesogenic pathways. Our findings elucidate a novel mechanistic pathway whereby age-related iron accumulation induces mitochondrial oxidative stress, which subsequently dysregulates energy balance through AgRP-mediated neural circuits.

Multiple studies have confirmed that oxidative stress-induced ROS activate the FoxO1 pathway, promoting its nuclear translocation and subsequent upregulation of target genes across metabolic tissues [50, 51]. In liver, ROS inhibit AKT phosphorylation, enabling nuclear FoxO1 to upregulate gluconeogenic (PEPCK, G6Pase) and lipogenic genes, exacerbating hyperglycemia and steatosis. In adipose tissue, ROS–FoxO1 signaling suppresses peroxisome proliferator-activated receptor γ (PPARγ) and promotes lipolysis (via adipose triglyceride lipase (ATGL)/hormone-sensitive lipase (HSL)) during fasting, but under overnutrition drives inflammation and insulin resistance via NF-κB/JNK [52, 53]. In muscle, FoxO1 activation by ROS downregulates glucose transporter type 4 (GLUT4) and induces atrophy programs (Atrogin-1/MuRF1), impairing glucose uptake and promoting sarcopenia [54, 55]. In pancreatic β-cells, ROS–FoxO1 inhibits pancreatic and duodenal homeobox 1 (PDX-1), impairing insulin secretion and promoting β-cells dedifferentiation [56, 57]. Here, we show that iron overload-induced ROS activate hypothalamic FoxO1, increasing AgRP expression and promoting age-dependent obesity, revealing a central role for this pathway in energy balance.

Hypothalamic iron accumulation promotes age-dependent obesity by increasing mitochondrial ROS and driving FoxO1 nuclear translocation, which upregulates AgRP expression and disrupts energy homeostasis. Our findings highlight the importance of age-related hypothalamic iron dysregulation in obesity pathogenesis and elucidate the impact of oxidative stress on AgRP neuronal function. Furthermore, this work provides novel insights for developing interventions against age-dependent obesity.

This study has several limitations. The relevance of hypothalamic iron accumulation in aging mice to humans remains unclear. All experiments used male mice, despite known sex differences in age-related weight gain [2, 58], including female subjects in future research would provide more comprehensive insights into iron's role in age-related metabolic homeostasis. Due to technical constraints, we were unable to specifically eliminate mitochondrial iron in AgRP neurons to determine whether metabolic changes result from mitochondrial iron overload. Although AgRP is also expressed in adrenal glands [59], the metabolic role of iron in these cells was not explored. Finally, glucose metabolic assessments relied solely on GTT and ITT, and may be confounded by body weight changes.

## Conclusions

In summary, this study elucidates the role and mechanism of hypothalamic iron accumulation in age-dependent obesity. We first identified an accumulation of iron in the hypothalamic ARC in aged mice. Employing both pharmacological and genetic approaches to remove iron from the hypothalamus, specifically from AgRP neurons, was found to increase energy expenditure and ameliorate age-dependent obesity and metabolic dysfunction. Mechanistic investigations revealed that iron overload induces mitochondrial dysfunction, leading to increased ROS production. The excess ROS promotes the nuclear translocation of FoxO1, which in turn upregulates AgRP expression, ultimately resulting in metabolic disorders. Our findings highlight the significance of hypothalamic iron dysregulation in age-dependent obesity and identify it as a promising novel therapeutic target.

## Method details

### Mice

Male C57BL/6 mice were supplied by Gem Pharmatech and maintained at 22–24 °C on a 12 h light/dark cycle with free access to food and water. Mice were fed a standard chow diet (9.4% kcal from fat; Xietong Bioscience).

The *AgRP-Cre* mice, *Tfrc*^*Loxp/Loxp*^ mice and *tdTomato reporter/Ai14* mice have been previously described [60]. *AgRP-Cre* mice were crossed with *Tfrc*^*Loxp/Loxp*^ mice to generate the mice with Tfrc specifically knockout in AgRP neurons (*AgRP-Cre*, *Tfrc*^*Loxp/Loxp*^). The *Tfrc*^*Loxp/Loxp*^ mice and *AgRP-Cre* mice were used as control. The *AgRP-Cre* mice were crossed with *tdTomato reporter/Ai14* mice to label the AgRP neurons. All mice used in this study were male mice.

### Measurement of metabolic parameters

The body composition of mice was assessed using a Minispec LF50 body composition analyzer (Bruker, Rheinstetten, Germany) and DEXA (InAlyzer, Seoul, South Korea). Oxygen consumption, carbon dioxide, EE and activity were assessed using a laboratory Animal Monitoring System (Columbus, St. Paul, MA, USA), and normalized by body weight.

### GTT and ITT

For glucose tolerance tests (GTT), mice fasted for 4–6 h or overnight were injected intraperitoneally with D-glucose (1.5 g/kg; Sigma, G8270) dissolved in saline. Blood glucose was measured at 0, 15, 30, 60, 90 and 120 min using a glucometer (Roche). For insulin tolerance tests (ITT), mice fasted for 4–6 h received an intraperitoneal injection of insulin (1.1 U/kg; Novo Nordisk, S20191007, 300 IU/3 mL). Blood glucose was monitored at the same time points.

### Treatment

For intranasal administration, aged mice were habituated as described^55^. Briefly, mice were gently restrained in a 45° position, and 5 μL saline was applied around the nares using a 10 µL micropipette, allowing complete inhalation between aliquots until 30 µL was delivered per session. This was repeated for three days prior to DFP treatment. On experimental days, DFP (3 mg/mL; MCE, HY-B0568) was administered intranasally at a total dose of 90 µg once daily for one month using the same procedure.

### Hypothalamic tissue harvesting

Mice were anesthetized with isoflurane and decapitated. The brain was rapidly extracted, and the hypothalamus was dissected using anatomical landmarks (optic chiasm anteriorly, mammillary bodies posteriorly, hypothalamic sulci laterally), then snap-frozen in liquid nitrogen for subsequent analysis.

### Biochemical parameters

Serum triglycerides (TG) were measured using a commercial assay kit (Jiancheng, A110-1–1). For hepatic TG, approximately 60 mg of liver tissue was homogenized in pre-chilled PBS with magnetic beads (60 Hz, 120 s). The supernatant was used for BCA protein quantification. Lipids were extracted from the homogenate with chloroform–methanol (2:1, v/v), centrifuged at 2000 × g for 10 min, and the lower organic phase was collected and dried. The residue was dissolved in 1% Triton X-100 in anhydrous ethanol before TG quantification using the same kit. Absorbance was measured at 500 nm.

Serum insulin was measured with an Ultra Sensitive Mouse Insulin ELISA Kit (Crystal Chem, #90,080) following the manufacturer’s low-range protocol. Absorbance was read at 450 nm with 630 nm as reference, and concentrations were determined from a standard curve.

### IF staining

Mice were anesthetized with avertin (300 mg/kg, i.p.) and transcardially perfused with 4% PFA. Brains were postfixed in 4% PFA for 4 h, cryoprotected in 20% and 30% sucrose, and sectioned at 25 μm using a cryostat (Leica CM1950). Sections were blocked in 5% serum/0.3% Triton X-100-PBS and incubated overnight at 4 °C with the following primary antibodies: anti-AgRP (Invitrogen, PA5-78,739, 1:200), anti-Ferritin (Abcam, ab75973, 1:100), anti-c-Fos (Synaptic Systems, 226,008, 1:1000), anti-Tfrc (Abclonal, A5865, 1:200), and anti-FoxO1 (CST, #2880, 1:400). After washing, sections were incubated with fluorescent secondary antibodies (Abcam: ab150106, ab150073; Invitrogen: A32814; all 1:1000) for 1 h at RT. Hypothalamic ROS was detected by DHE staining [58]. Images were acquired on a Zeiss LSM980 confocal microscope and analysed using Image-Pro Plus 6.0 software.

### Hematoxylin and eosin (H&E) staining and Oil Red O staining

For H&E staining, eWAT, sWAT and liver tissues were fixed in 4% PFA, embedded in paraffin and sectioned at a thickness of 5 μm. The sections were stained with H&E. For Oil Red O staining, tissues were sectioned using a cryostat. Images were captured using a Leica DM4 B microscope (Leica, Buffalo Grove, USA). The areas were analyzed using Image-Pro Plus (Version 6; Media Cybernetics, Rockville, MD, USA), counted the number of fat cells in the same range of vision in each mouse.

### Quantitative real-time PCR

Total RNA was extracted using the RNeasy Plus Universal Mini Kit (Qiagen, 73,404). cDNA was synthesized from 1 μg RNA with PrimeScript™ RT Master Mix (TaKaRa, RR036A). Quantitative PCR was performed using SYBR Green Premix (Thermo Fisher) on a QuantStudio 7 Flex system. Relative expression was determined using the 2^−△Ct^ method normalized to Gapdh. Primer sequences are provided in Table S1.

### Western blot

Total protein was extracted using lysis buffer (30 mM HEPES, 150 mM NaCl, 1% Triton X-100, 10% glycerol) supplemented with protease and phosphatase inhibitors (Roche). Proteins were separated by SDS-PAGE (Bio-Rad) and transferred to nitrocellulose membranes (Millipore). After blocking with 5% non-fat milk, membranes were incubated overnight at 4 °C with the following primary antibodies: anti-ferritin (Cell Signaling Technology, 4393, 1:1000), anti-FoxO1 (Cell Signaling Technology, 2880, 1:1000), anti-NDUFB8 (Proteintech, 14,794–1-AP, 1:5000), anti-SDHB (Proteintech, 10,620–1-AP, 1:5000), anti-UQCRC2 (Proteintech, 14,742–1-AP, 1:2000), anti-MTCO2 (Proteintech, 55,070–1-AP, 1:2000), anti-ATP5A1 (Proteintech, 14,676–1-AP, 1:5000), and anti-β-actin (Cell Signaling Technology, 4967, 1:2000). Following incubation with HRP-conjugated secondary antibody (Cell Signaling Technology, 7074, 1:5000) and extensive washing with PBST, bands were visualized using an enhanced chemiluminescence substrate (Omni-ECL™, Epizyme SQ201) and imaged with a ChemiDoc system (Bio-Rad). Quantification was performed using ImageJ software (v1.52, NIH). Protein levels were normalized to β-actin.

### Determining the hypothalamic iron concentration

Hypothalamic iron content was measured using an Iron Assay Kit (Abcam, ab83366) per manufacturer’s instructions. Tissue was homogenized in assay buffer, digested on ice, and centrifuged at 12,000 × g for 10 min at 4 °C. The supernatant was incubated with iron reducer for 30 min at RT, followed by addition of iron probe and shaking for 60 min. Absorbance was read at 593 nm using a SpectraMax 190 Microplate Reader (Molecular Devices). Iron levels were normalized to total protein.

### DAB-enhanced Perls' Prussian blue staining

Ferric iron (Fe^3^⁺) was detected using DAB-enhanced Perls’ Prussian blue staining. Brain sections were treated with protease K (30 µg/mL) in 0.1% Triton X-100/PBS for 20 min at 37 °C, followed by 1% H₂O₂ for 5 min at RT. Sections were then incubated in 2% potassium ferrocyanide in 0.125 N HCl with 1% Triton X-100 for 30 min at 37 °C, developed in DAB for 3 h at 37 °C, dehydrated, cleared, and mounted. Imaging was performed using a Zeiss LSM 980 confocal microscope. Iron deposition was quantified by optical density analysis with ImageJ (v1.8, NIH).

### Cell culture

GT1-7 cells were maintained in DMEM with 10% FBS and 1% penicillin–streptomycin at 37 °C under 5% CO₂ and 20% O₂. Cells were treated for 24 h with 200 μM FAC (Sigma, F3388), 200 μM NAC (MCE, HY-B0215), 10 μM Mito-TEMPO (MCE, HY-112879), or 5 μM AS1842856 (MCE, HY-100596).

Cellular ATP levels were measured using an Enhanced ATP Assay Kit (Beyotime, S0027). Lysates were centrifuged at 12,000 × g for 5 min at 4 °C, and luminescence of the supernatant was measured with a SpectraMax M2 microplate reader (Molecular Devices).

Mitochondrial membrane potential was assessed using the JC-1 Assay Kit (Beyotime, C2003S). Live cells were stained with JC-1 for 20 min at 37 °C in the dark. Fluorescence was imaged with a Nikon C2si confocal microscope and quantified red/green ratios were obtained using a SpectraMax M2 reader.

### Mitochondrial iron staining

Cells were cultured under standard conditions (37 °C, 5% CO₂). After washing with serum-free medium, cells were incubated with 5 μmol/L Mito-FerroGreen (DOJINDO, M489) for 30 min at 37 °C, followed by additional washes and treatment with FAC. Imaging was performed using a Zeiss LSM 980 confocal microscope. Iron intensity was quantified via optical density analysis using ImageJ (v1.8, NIH).

### Malondialdehyde (MDA) quantification

Cellular MDA levels were measured using a Lipid Peroxidation Assay Kit (Beyotime, S0131S). Cells were lysed and centrifuged at 12,000 × g for 10 min at 4 °C. The supernatant was incubated with MDA working solution at 100 °C for 15 min, cooled, and centrifuged at 1000 × g for 10 min. Absorbance was measured at 532 nm using a SpectraMax M2 microplate reader (Molecular Devices). MDA levels were normalized to total protein content.

### Statistical analysis

All data was presented as the mean ± SEM. Statistical analyses were performed using Prism 9 (GraphPad Software, San Diego, CA, USA). For two-group comparisons, a two-tailed Student's t-test was utilized. two-way ANOVA with Bonferroni's post hoc test were applied for comparisons among more than two groups. ACNOVA was applied to assess the influence of body weight on EE. Statistical significance was set at *p* < 0.05.

## Supplementary Information

Supplementary Material 1.

## Data Availability

The corresponding author will provide the original data used to support the findings of this study upon reasonable request.
