# Supplementary material for: Iron accumulation in hypothalamus promotes age-dependent obesity and metabolic dysfunction of male mice
Source: Mol Biomed. 2025 Oct 2;6:75. doi: 10.1186/s43556-025-00324-0 (PMC12491144; doi:10.1186/s43556-025-00324-0)
Supplement: Supplementary file 1 — Supplementary Material 1. [file 43556_2025_324_MOESM1_ESM.docx]

**Iron accumulation in hypothalamus promotes age-dependent obesity and metabolic dysfunction of male mice**

Xinyu Wang^1, 2†^, Xiaoyue Xiong^1†^, Ye Xuan^1†^, Wen Tian^1, 3^, Liwei Chen^1^, Zhuo Chen^1*^, Yi Zhang^1*^, Wei L. Shen^2*^, Cheng Hu^1,3 *^

**Affiliations**

^1^Shanghai Diabetes Institute, Shanghai Key Laboratory of Diabetes Mellitus, Shanghai Clinical Centre for Diabetes, Clinical Research Center, Shanghai Sixth People's Hospital Affiliated to Shanghai Jiao Tong University School of Medicine, Shanghai 200233, China.

^2^School of Life Science and Technology, ShanghaiTech University, Shanghai 201210, China.

^3^Department of Endocrinology, Jinzhou medical university, Jinzhou 121001, China.

^†^These authors contributed equally.

^*^Corresponding author. [zhuochen_tys0110@sjtu.edu.cn](mailto:zhuochen_tys0110@sjtu.edu.cn) (C.Z); yi.zhang@sjtu.edu.cn (Y.Z); [shenwei@shanghaitech.edu.cn](mailto:shenwei@shanghaitech.edu.cn) (W.L.S); alfredhc@sjtu.edu.cn (C.H)


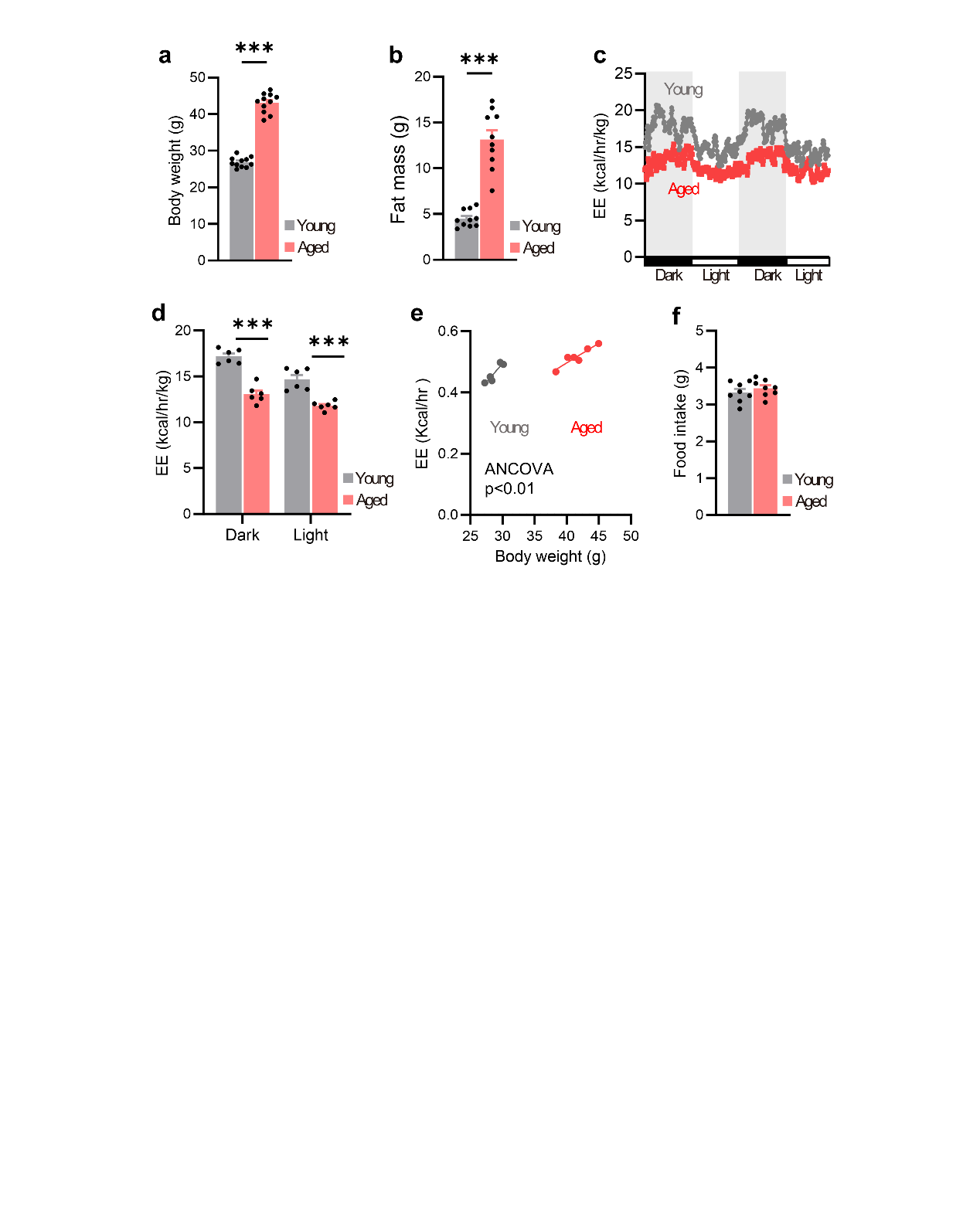


**Figure S1. Comparative assessment of metabolism function in aged mice versus young mice**

(a) Body weight of young and aged mice (n = 11 for each group).

(b) Fat mass of young and aged mice (n = 10 for each group).

(c-e) EE of young and aged mice (n = 6 for each group).

(f) Cumulative food intake during 24 hours of young and aged mice (n = 8 for each group).

Data was represented as mean ± SEM. **p* < 0.05, ***p* < 0.01, ****p* < 0.001, two-tailed Student’s t-test (a, b, d and f), ANCOVA (e).


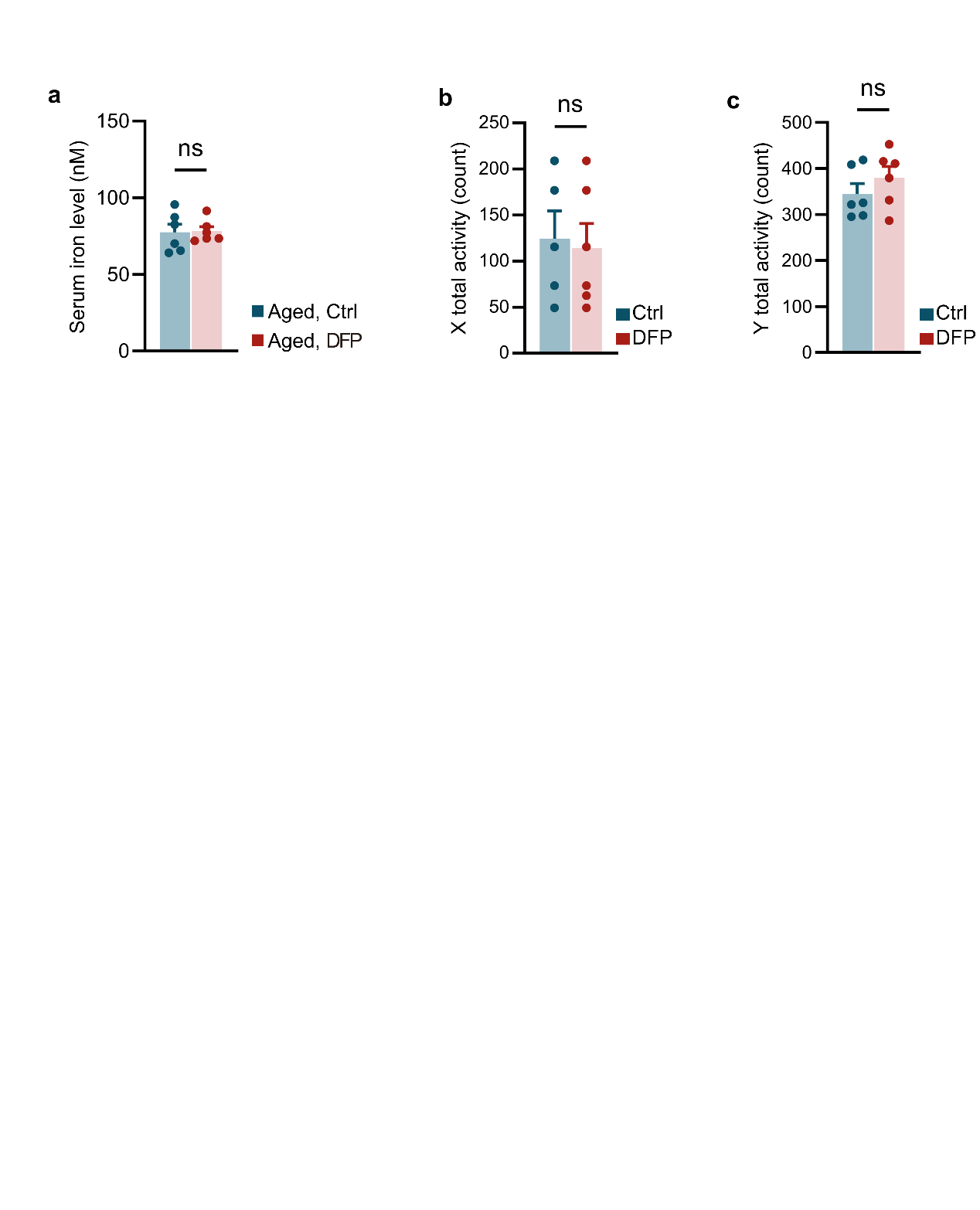


**Figure S2. Supplementary metabolism function in aged mice received one month of intranasal administration of saline or DFP**

(a) Serum iron levels of aged mice received one month of intranasal administration of DFP or saline (n = 6 for each group DFP).

(b, c) Total activity of aged mice received one month of intranasal administration of DFP or saline measured by monitoring system (n = 5 for Ctrl, n = 6 for DFP).

Data was represented as mean ± SEM. **p* < 0.05, ***p* < 0.01, ****p* < 0.001, two-tailed Student’s t-test (a-c).


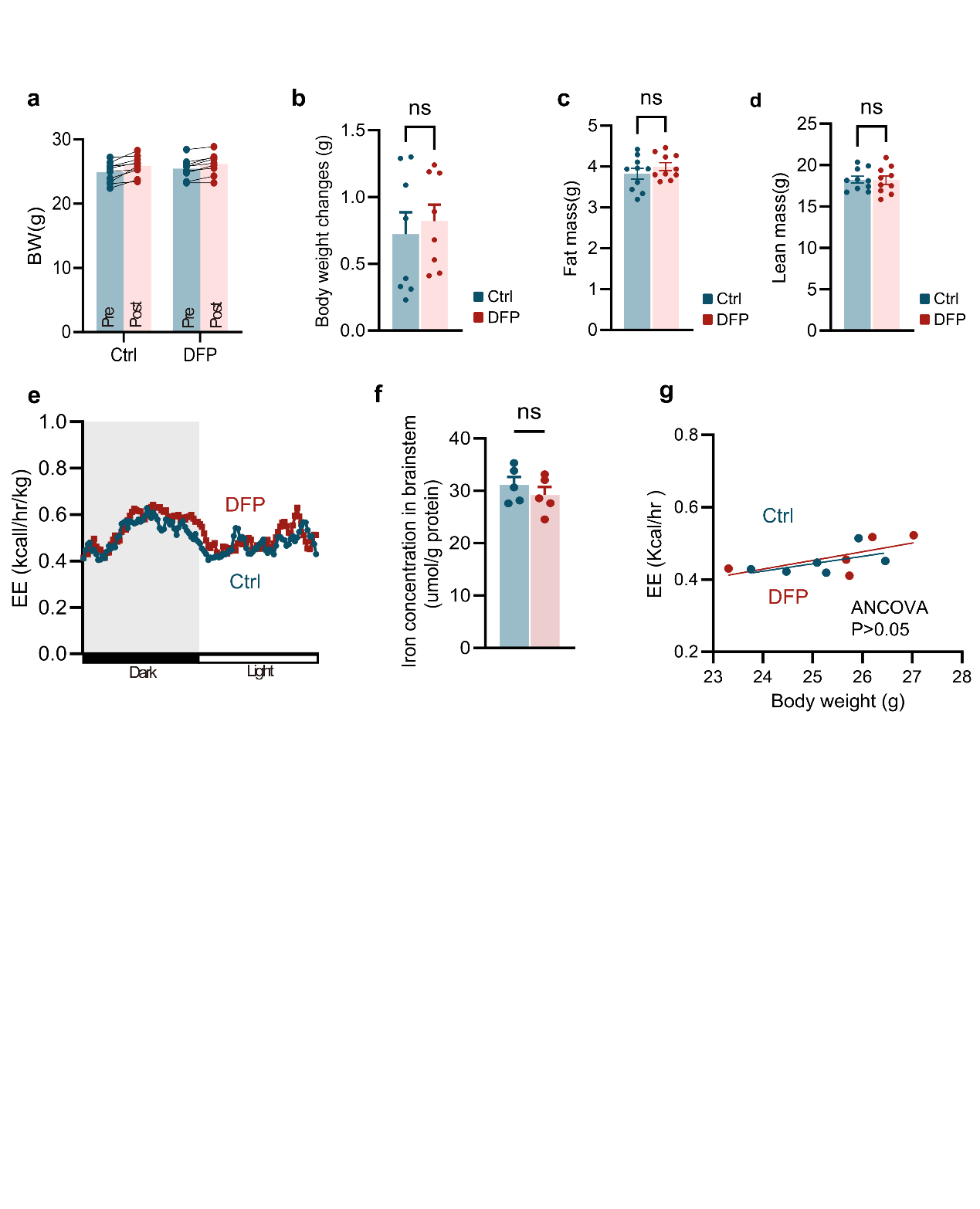


**Figure S3. Metabolism function of young mice received one month of intranasal administration of saline or DFP**

(a, b) Body weight (a) and body weight changes (b) of young mice received one month of intranasal administration of DFP or saline (n = 8 for each group).

(c, d) Fat mass (c) and lean mass (d) of young mice received one month of intranasal administration of DFP or saline (n = 10 for each group).

(e-g) EE of young mice received one month of intranasal administration of DFP or saline (n = 6 for Ctrl, n = 5 for DFP).

Data was represented as mean ± SEM. **p* < 0.05, ***p* < 0.01, ****p* < 0.001, two-tailed Student’s t-test (b, c, d and f), two‐way ANOVA with Bonferroni’s post hoc test (a), ANCOVA (g).


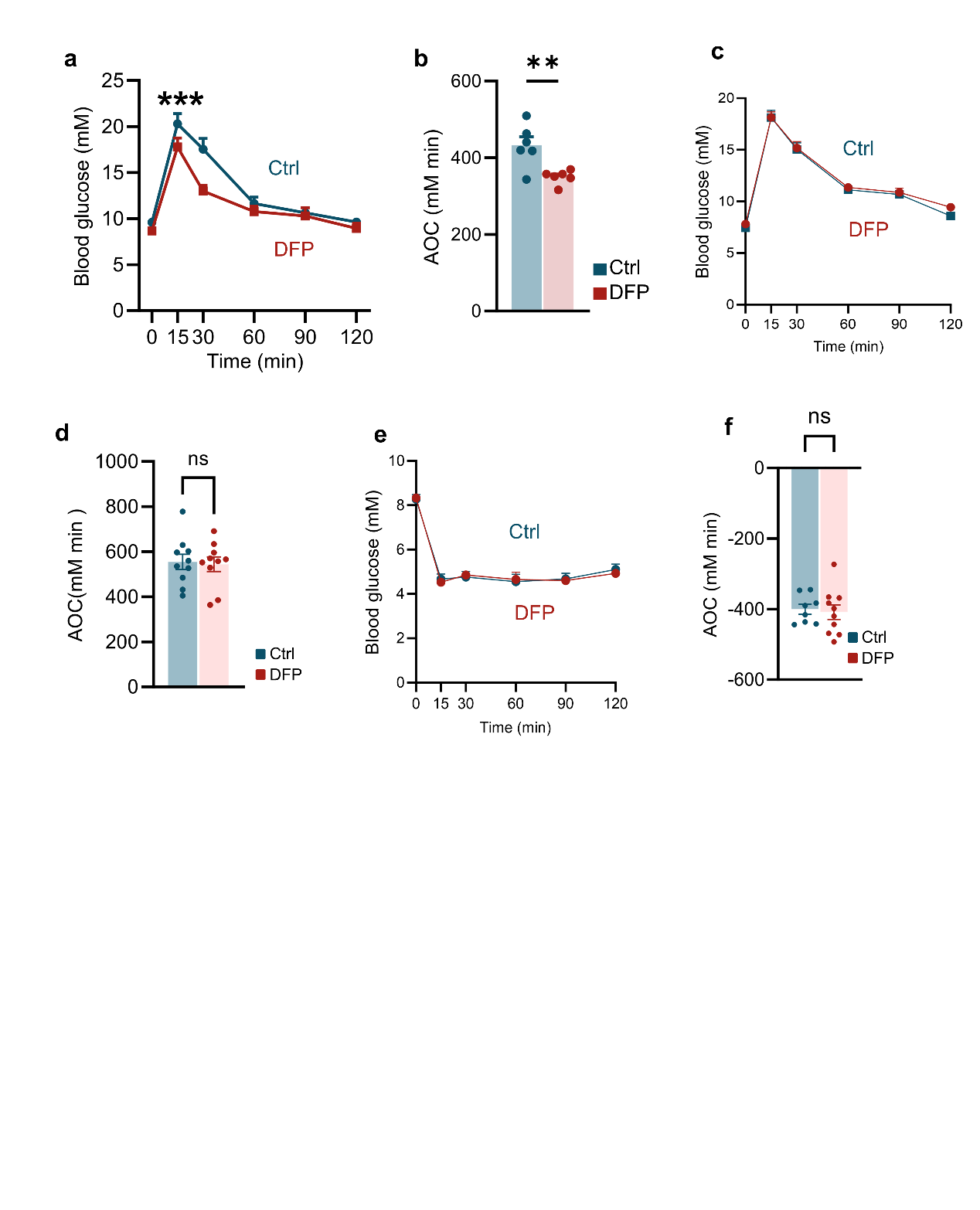


**Figure S4. Supplementary glucose tolerance and insulin tolerance of mice received one month of intranasal administration of saline or DFP**

(a, b) GTT (a) and AOC of GTT (b) of aged mice received one month of intranasal administration of DFP or saline fasted for 5-6 hours. Glucose was administered at doses adjusted for lean mass (n = 6 for each group).

(c, d) GTT (c) and AOC of GTT (d) of young mice received one month of intranasal administration of DFP or saline (n = 10 for each group).

(e, f) ITT (e) and AOC of ITT (f) of young mice received one month of intranasal administration of DFP or saline (n = 8 for Ctrl, n = 10 for DFP).

Data was represented as mean ± SEM. **p* < 0.05, ***p* < 0.01, ****p* < 0.001, two-tailed Student’s t-test (b, d and f), two‐way ANOVA with Bonferroni’s post hoc test (a, c and e).


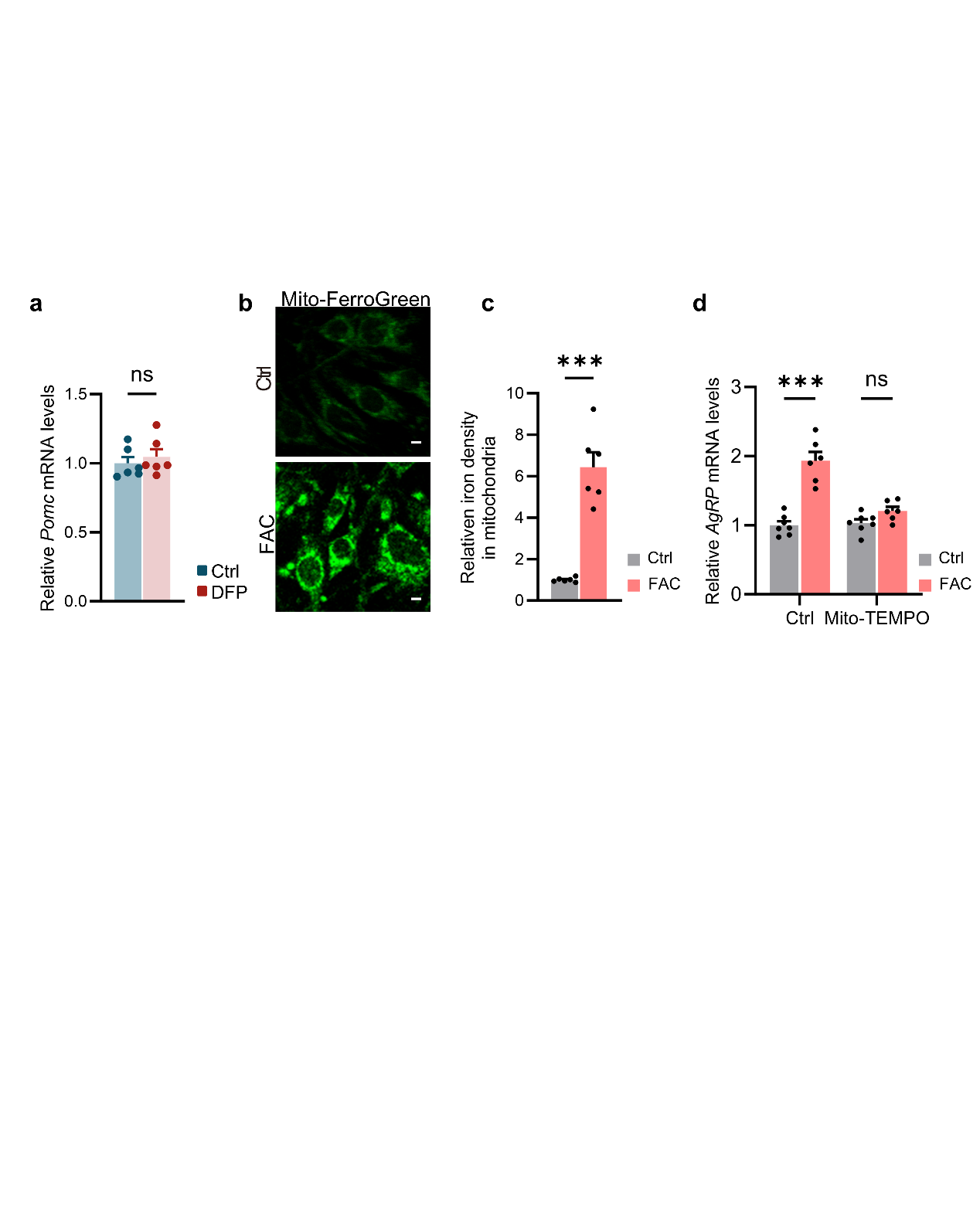


**Figure S5. Supplemental characterization of iron overload regulated the expression of AgRP by promoting mitochondrial oxidative stress**

(a) Relative mRNA levels of *Pomc* in the hypothalamus of aged mice following one month of intranasal administration of DFP or saline (n = 6 for each group), as measured by RT–qPCR.

(b, c) Representative Mito-FerroGreen staining (a) and fluorescence intensity quantification (b) in GT1-7 cells treated with FAC (n = 6 for each group).

(d) Relative mRNA levels of Agrp in GT1-7 cells treated with FAC or Ctrl, in the presence or absence of Mito-TEMPO (n = 7 for Ctrl, n = 6 for FAC).

Data was represented as mean ± SEM. **p* < 0.05, ***p* < 0.01, ****p* < 0.001, two-tailed Student’s t-test (a and c). two‐way ANOVA with Bonferroni’s post hoc test (d). Scale bar, 50 μm.


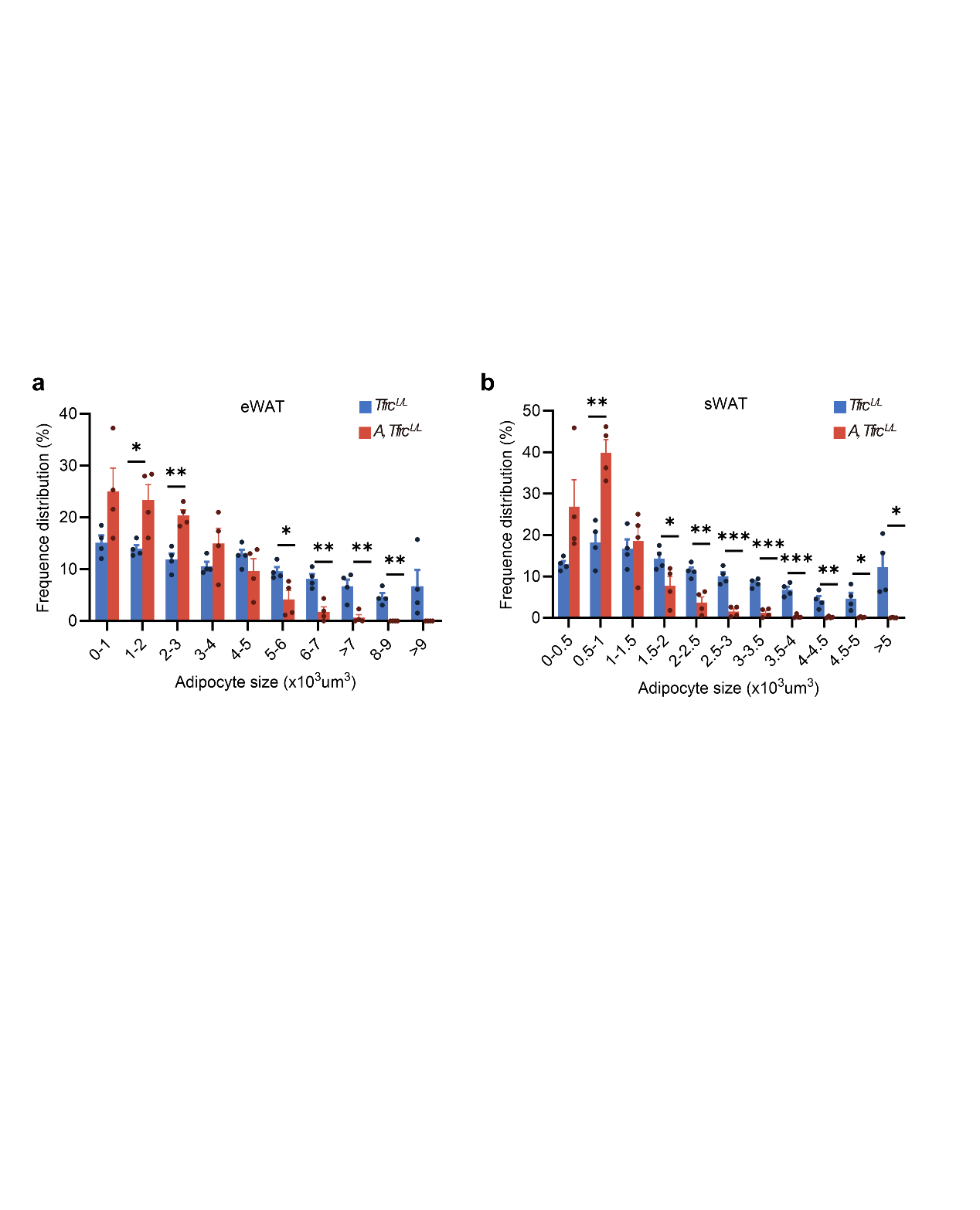


**Figure S6. Adipocyte size distribution of mice with TFRC ablation in AgRP neurons**

(a, b) Adipocyte size distribution in eWAT (a) and sWAT (b) of *Tfrc^L/L^* and *A, Tfrc^L/L^* mice fed a CD for 16 months (n = 4 for each group).

Data was represented as mean ± SEM. **p* < 0.05, ***p* < 0.01, ****p* < 0.001, two-tailed Student’s t-test.

**Table S1 Oligonucleotide primers for quantitative RT-PCR analysis.**

| Gene | Forward primer (5′-3′) | Reverse primer (5′-3′) |
| --- | --- | --- |
| β-Actin | GTCCACCCCGGGGAAGGTGA | AGGCCTCAGACCTGGGCCATT |
| Srebf1 | TGACCCGGCTATTCCGTGA | CTGGGCTGAGCAATACAGTTC |
| Hmgcr | TGTTCACCGGCAACAACAAGA | CCGCGTTATCGTCAGGATGA |
| Acc1 | ATGGGCGGAATGGTCTCTTTC | TGGGGACCTTGTCTTCATCAT |
| Acl | TGTTCTTGGTCAGCTTTGTAGC | AGGCTGTGGGTCTTGTTTAGG |
| Me1 | GTCGTGCATCTCTCACAGAAG | TGAGGGCAGTTGGTTTTATCTTT |
| Gsk3b | ATGGCAGCAAGGTAACCACAG | TCTCGGTTCTTAAATCGCTTGTC |
| G6pc | CGACTCGCTATCTCCAAGTGA | GGGCGTTGTCCAAACAGAAT |
| Agrp | TTGTGTTCTGCTGTTGGCACT | ATCTAGCACCTCCGCCAAAG |
